# Dataset on customer experience and satisfaction in healthcare sector of Nigeria

**DOI:** 10.1016/j.dib.2018.06.070

**Published:** 2018-07-05

**Authors:** Taiye Borishade, Oladele Kehinde, Oluwole Iyiola, Maxwell Olokundun, Ayodotun Ibidunni, Joy Dirisu, Charles Omotoyinbo

**Affiliations:** Covenant University, Nigeria

**Keywords:** Customer, Experience, Customer satisfaction, Healthcare, Nigeria

## Abstract

The central aim of the study was to show a dataset that empirically examines the connection between customer experience (CE) and customer satisfaction (CS). Few or no research have investigated how customer experience can be used to improve customer satisfaction in the healthcare sector of Nigeria. The study therefore adopted a survey research and the data were generated via a structured questionnaire. A total of 365 copies of the questionnaire were retrieved from the customers of the selected four private hospitals in Lagos State. The questionnaire administered were analysed using SPSS (version 22). Using the descriptive and the Categorical Regression CATREG analysis, the data explained how customer experience have a significant relationship with customer satisfaction. The data gathered is provided openly so as to facilitate further analysis

**Specification Table**

**Table:** 

**Subject area**	Marketing
**More Specific Subject Area:**	Healthcare Marketing
**Type of Data**	Primary data, Tables
**How Data was Acquired**	Field Survey
**Data format**	Raw data and analyzed
**Experimental Factors**	Survey sample comprised customers in the selected private hospitals in Lagos State, Nigeria.
**Experimental features**	The respondents surveyed responded to the research instrument on the influence of customer experience on customer satisfaction
**Data source location**	Lagos State, Nigeria
**Data Accessibility**	Data are contained within this study.

**Value of data**

• Comprehensive customer experience data presented will stimulate support based research in the growing field of customer experience in developing nations.
• The dataset provided in this data article describes customer experience and customer satisfaction in healthcare service delivery, which could be an eye-opener for other researchers to extend the statistical study.
• The data presented will inspire empirical research that would help to measure the present trends in healthcare services and how the delivery of quality healthcare services could be enhanced by practitioners.

## 1 Data

The data is the result of a survey on customer experience and customer satisfaction in the healthcare sector of Nigeria. The data present on a 5-linkert scale, the mean score and the ranking of the respondents based on their opinion on the connection between customer experience and satisfaction. In order to test for the trend and strength of the relationships between the two variables, Categorical Regression (CATREG) analysis was employed to find out the degree of relationship that exists between customer experience and satisfaction (Table 1).

**Table 1:** The relationship between customer experience and customer satisfaction.

**Components of CE and CS**	***N***	**Mean(Satisfaction score)**	**Std. Deviation**	**Rank**
	**Statistic**	**Statistic**	**Statistic**	
Showing professionally appropriate behaviour	365	4.17	0.825	1
Maintaining patient privacy	365	4.17	0.881	1
Right treatment of illness	365	4.16	0.771	2
Reliability of the health care services.	365	4.12	0.838	3
Cleanliness of the health care organisation	365	4.10	0.881	4
Right diagnosis of illness	365	4.08	0.806	5
Effective verbal communication.	365	4.06	0.864	6
Respecting my wishes	365	3.96	0.953	7
Aroma of the health care organization	365	3.75	1.150	8
Non-verbal communication.	365	3.66	1.074	9
Valid N (list wise)	365			

## 2 Test of hypothesis

H_0_ : There is no significant connection between customer experience and customer satisfaction.

Table 2: The *R* Square tells the extent of the moderation in the dependent variable (customer satisfaction) is described by the model (Customer experience: The reliability of services; the right diagnosis of illness; the right treatment of illness; the effective verbal communication and the non-verbal communication). Here, the *R* Square value 0.431 is stated as a percentage; this means that the model (Customer experience) explains 43.1% of the variance on customer satisfaction. This is very reasonable result when compare to some outcome that are reported in the journals [1], [2], [4].

**Table 2:** Model summary of the relationship between customer experience and satisfaction.

Multiple *R*	*R* square	Adjusted *R* square	Apparent prediction error
0.656	0.431	0.408	0.569

Predictors: The reliability of the health care services; the right diagnosis of illness by the health care organisation; the right treatment of illness by the health care organisation; the effective verbal communication of the health care service and he non-verbal communication of the health care service.

Dependent Variable: Customer satisfaction.

The ANOVA of the regression model is also presented Table 3. It is evident from the result (Table 3) that the regression model has F (14,350)=18.920 is significant with *P*<0.0005. This means that the null hypothesis (H_0_) is rejected as we accept the alternate hypothesis (Table 4).

**Table 3:** ANOVA of the relationship between customer experience and satisfaction.

	Sum of squares	Df	Mean square	*F*	Sig.
Regression	157.237	14	11.231	18.920	0.000
Residual	207.763	350	0.594		
Total9	365.000	364

**Table 4:** Coefficients of regression analysis of the relationship between customer experience and satisfaction.

	Standardized Coefficients		Df	F	Sig.
	Beta	Bootstrap (1000) Estimate of Std. Error			
The reliability of the health care services.	0.229	0.073	3	9.708	0.000
The right diagnosis of illness by the health care organisation.	0.258	0.075	4	11.915	0.000
The right treatment of illness by the health care organisation.	0.106	0.111	2	.911	0.403
The effective verbal communication of the health care service provider.	0.161	0.078	2	4.274	0.015
The non-verbal communication of the health care service provider.	0.196	0.060	3	10.780	0.000

Dependent Variable: Customer satisfaction.

## 3 Experimental design, materials and methods

The data for this study are quantitative in nature. The quantitative data are mainly scores in variables used to measure customer experience and satisfaction with healthcare services. Data were derived from the customers (patients) of the selected private healthcare organisations in Lagos, Nigeria. The survey research design was used to gather data from 365 respondents from four private hospitals. The connection between customer experience and customer satisfaction of respondents was investigated by asking them to indicate the magnitude of their agreement with the questions related to customer experience and satisfaction on a 5-point Likert scale, where 5=strongly agree, 4=agree, 3=undecided, 2=disagree and 1=strongly disagree. The data collected from the respondents were subjected to descriptive statistics including the computation of the sum, mean, standard deviation [2]. The categorical regression analysis with optimal scaling technique otherwise regarded as CATREG in SPSS was also used to investigate which aspect of the customer experience influence customer satisfaction with healthcare services in the four hospitals investigated [4]. Ethical consideration was ensured because responding to the questionnaires was centered on the willingness of the customers (patients) to respond [3].
